# Electromagnetic tracking (EMT) technology for improved treatment quality assurance in interstitial brachytherapy

**DOI:** 10.1002/acm2.12021

**Published:** 2017-01-19

**Authors:** Markus Kellermeier, Jens Herbolzheimer, Stephan Kreppner, Michael Lotter, Vratislav Strnad, Christoph Bert

**Affiliations:** ^1^ Department of Radiation Oncology Universitätsklinikum Erlangen Friedrich‐Alexander‐Universität Erlangen‐Nürnberg Universitätsstraße 27 91054 Erlangen Germany

**Keywords:** catheter reconstruction, EM tracking, error detection, HDR brachytherapy, quality assurance

## Abstract

Electromagnetic Tracking (EMT) is a novel technique for error detection and quality assurance (QA) in interstitial high dose rate brachytherapy (HDR‐iBT). The purpose of this study is to provide a concept for data acquisition developed as part of a clinical evaluation study on the use of EMT during interstitial treatment of breast cancer patients. The stability, accuracy, and precision of EMT‐determined dwell positions were quantified. Dwell position reconstruction based on EMT was investigated on CT table, HDR table and PDR bed to examine the influence on precision and accuracy in a typical clinical workflow. All investigations were performed using a precise PMMA phantom. The track of catheters inserted in that phantom was measured by manually inserting a 5 degree of freedom (DoF) sensor while recording the position of three 6DoF fiducial sensors on the phantom surface to correct motion influences. From the corrected data, dwell positions were reconstructed along the catheter's track. The accuracy of the EMT‐determined dwell positions was quantified by the residual distances to reference dwell positions after using a rigid registration. Precision and accuracy were investigated for different phantom‐table and sensor‐field generator (FG) distances. The measured precision of the EMT‐determined dwell positions was ≤ 0.28 mm (95th percentile). Stability tests showed a drift of 0.03 mm in the first 20 min of use. Sudden shaking of the FG or (large) metallic objects close to the FG degrade the precision. The accuracy with respect to the reference dwell positions was on all clinical tables < 1 mm at 200 mm FG distance and 120 mm phantom‐table distance. Phantom measurements showed that EMT‐determined localization of dwell positions in HDR‐iBT is stable, precise, and sufficiently accurate for clinical assessment. The presented method may be viable for clinical applications in HDR‐iBT, like implant definition, error detection or quantification of uncertainties. Further clinical investigations are needed.

## 1 Introduction

Interstitial brachytherapy (iBT) is an established treatment option for treating cancer in numerous body sites including, e.g., prostate,^1^ head & neck,^2^ breast,^3^ and gynecology.^4^ For breast treatments accelerated partial breast irradiation (APBI) is proven to have at least comparable outcome to established whole breast treatment schemes.^5^, ^6^, ^7^

In current clinical practice, catheter positions are reconstructed based on computed tomography (CT), magnetic resonance tomography (MRT) or ultrasound imaging data.^8^ The accuracy of the reconstructed catheters depends on the imaging modality, but can be hampered by imaging artifacts, the limited slice thickness of CT/MRT data which typically ranges from 2 to 5 mm for brachytherapy planning, the individual anatomy of the patient, the treatment site, or approximations such as deviations between the reconstructed and the actual source path that can lead to discrepancies up to 5.5 mm.^9^ In addition, the current clinical procedures for implant reconstruction are time‐consuming and observer‐dependent.^10^

In parallel to the clinical success of brachytherapy, efforts are increasing to address and reduce uncertainties and to detect and avoid errors in brachytherapy treatments.^9^, ^11^, ^12^ Like in other high precision therapy options such as stereotactic (external beam) radiation therapy, for which, e.g., intensity modulated treatment plans are verified in dosimetry phantom before treatment,^13^ these errors could very likely be identified prior treatment. Among the most common medical events reported by the Nuclear Regulatory Commission (NRC) in the United States related to high‐dose‐rate (HDR) treatment planning are: wrong indexer length, catheter reconstruction errors, and misidentified first dwell position.^11^ One option for verifying the 3D implant geometry and the corresponding dwell positions is electromagnetic tracking (EMT) as described in the following.

EMT with miniaturized electromagnetic sensors is widely used in clinical practice. Examples are tracking of instruments in surgical interventions,^14^ guidance of biopsies,^15^ and motion monitoring in ablation 16 or external beam radiation therapy.^17^ A review of the technological fundamentals and the clinical applications is given by Franz et al.^18^ In the last years, EMT was also proposed for interstitial brachytherapy mainly addressing implant reconstruction and error detection,^19^ i.e., applications in‐line with the ongoing efforts to increase quality assurance (QA) in brachytherapy.

Several studies in phantoms exploring the use of EMT in HDR‐iBT have focused on implant reconstruction.^10^, ^20^, ^21^ Zhou et al. addressed HDR treatments of prostate cancer with the goal to increase the accuracy and speed of the implant tracking compared to ultrasound images based reconstruction as the current clinical standard.^20^ Using a calibration phantom, they assessed the noise level and tracking accuracy in their operating room, including the known interferences of EMT and (ferromagnetic) metals in the clinical environment. They report an accuracy of 1.6 ± 0.2 mm in the operating room, supporting the findings of Nixon et al.^22^ that especially the distance of the field generator to the sensor should be minimized. The authors concluded that EMT‐based implant reconstruction is faster and more accurate than the ultrasound‐based procedure. Similar conclusions have been drawn by Bharat et al.,^10^ who reported agreement in EMT, CT, and TRUS‐identified implant geometries, and Poulin et al.,^21^ who found that EMT based reconstruction of the implant is more accurate than CT.

The focus is shifted toward EMT‐based error detection for HDR‐iBT in the work of Damato et al.^23^ In a phantom study, they mimicked swapping, wrong intersecting, and shifting of catheters and tried to identify the introduced errors by EMT. Identification of catheter swapping and wrong intersections was successful in all studied cases. Shifts could be identified with 100% sensitivity and specificity if their magnitude was > 2.6 mm.

We aim to assess the clinical feasibility of error detection and monitoring of the implant geometry by EMT in interstitial HDR breast cancer treatments. For interstitial multicatheter brachytherapy after breast‐conserving surgery, the standard HDR treatments at the University Clinic Erlangen, Germany are currently planned using CT‐based implant reconstruction and delivered in nine fractions within 5 days.

The purpose of this study was to provide a technical description of the data acquisition and processing procedure that was developed as part of a clinical evaluation study on the use of EMT during interstitial treatment of breast cancer patients. The procedure includes a protocol for compensation of breathing motion that influences the catheter EMT measurements. Based on the data acquisition technique the stability, accuracy, and precision of EMT‐determined dwell positions were quantified.

## 2 Material and methods

### 2.A The EMT system

**Figure 1:**
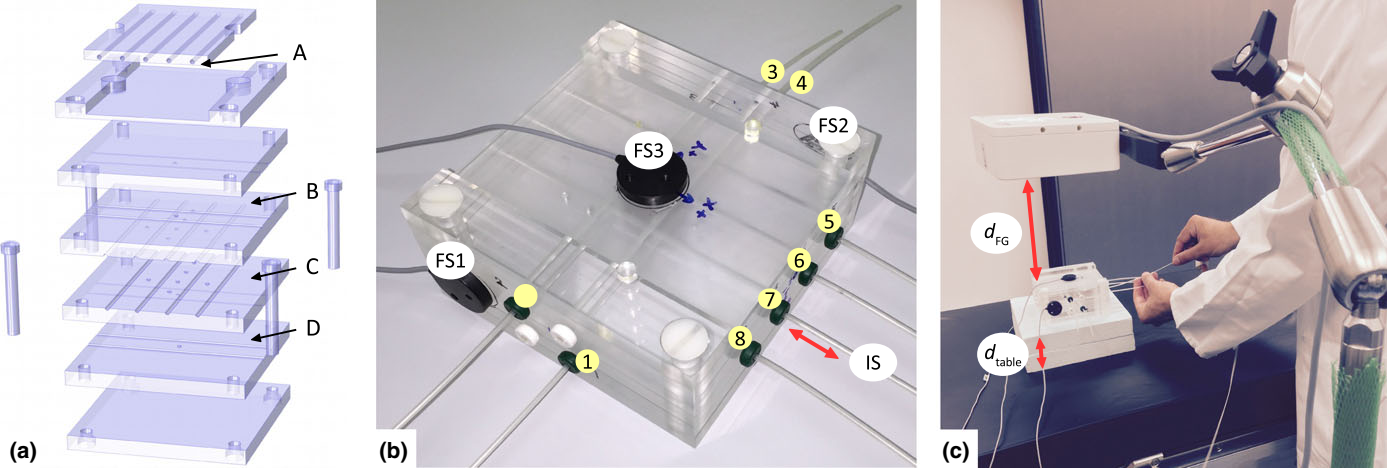
(a) Exploded view drawing of our quality cross‐check phantom (Section 2.B) made out of transparent PMMA plates designed for position checks of cables with an active source (A) and precise guiding of catheters at different heights and orientations (B‐D). Several spherical air pockets can be used for image‐based QA and as landmarks for registration, e.g. with CT‐images of the phantom. (b) Picture of the phantom (120 × 120 × 60 mm^3^) with plastic catheters (*i* = 1, …, 8), three fixed fiducial sensors (FS) and an implant sensor (IS) in catheter (*i* = 7). (c) Measurement setup on the HDR treatment table. The distances *d*_table_ and *d*_FG_ were varied to study their influence on precision and accuracy.

In this study, the third generation Aurora^®^ Electromagnetic Tracking System from Northern Digital Inc. (NDI, Waterloo, Canada) was used. The system's field generator (FG) was mounted on a flexible and lockable position arm over the phantom placed on a patient bed, scanner table or treatment table (Fig. 1). The FG covers a cubic tracking field of 500 × 500 × 500 mm^3^. The positions of four sensors were tracked at the maximum measuring rate of 40 Hz. In this study we had interest in the positions, **p** = (x, y, z) in $R^{3}$ , of one 5 degree of freedom (DoF) sensor (Aurora 5DoF Catheter, Type 1, 1.2 mm diameter) in a catheter (= implant sensor, IS) and in addition of three 6DoF sensors (Aurora 6DoF reference, 25 mm disc, standard) fixed on the object's surface (here on the phantom, Section 2.B) to obtain fiducials (= fiducial sensors, FS). Both sensor types are solenoid search coils from NDI. A single 5DoF sensor measures translation (3DoF) and the two rotations perpendicular to its longitudinal axis. 6DoF sensors are made up from two 5DoF sensors. For the measurements reported in this study, a single set of 6DoF sensors and two different 5DoF sensors were used.

The Aurora's system units were placed on a trolley together with a laptop computer. Based on the Aurora API interface guide (Revision 4) a MATLAB (The Mathworks, Natick, MA, USA) application was developed and verified for the upcoming HDR breast treatment study. With our software, we were able to record the EMT data received via serial communication, to interpret it in real‐time, and to simultaneously carry out the initial data analysis routines.

### 2.B The quality cross‐check phantom

To assess the precision and accuracy of the EMT system in its clinical brachytherapy mode of operation, we constructed a phantom that will also be used for regular QA purposes (see Fig. 1). The design is inspired by the so‐called Baltas phantom.^24^ In this study, we only used the geometric catheter configuration of our phantom.

The 120 × 120 × 60 mm^3^ phantom is built out of six polymethyl methacrylate (PMMA) plates (each with a height of 20 mm). A total of eight plastic catheters (6F Flexible Implant Tubes, Elekta Brachytherapy Veenendaal, The Netherlands) are linearly guided by grooves of the aligned and fixed PMMA plates (see Fig. 1). Four of the catheters are centrally arranged between the middle plates at a (center‐to‐center) distance of 20 mm from each other to obtain the usual pitch of the holes in the template used for breast implants. Parallel to this level at a right angle (90° and −90°), each 20 mm above and below, two catheters are placed at a distance of 40 mm to each other. The manufactured phantom dimensions were measured by a calibrated caliper (± 0.01 mm) and formed the basis for the reference dwell positions (Section 2.D).

The phantom was also used to determine the measuring location of the implant sensor relative to its physical tip [used as distance $\delta_{\mathrm{sensor}}$. see Eq. (1)]. By approaching the sensor from opposite directions to the outer walls of the phantom the known dimension $d_{\mathrm{nom}}$. the phantom can be compared with the distance $d_{\mathrm{EMT}}$_._ termined by EMT, yielding $\delta_{\mathrm{sensor}}$ via:

(1) $$\delta_{\mathrm{sensor}}=\left(d_{\mathrm{EMT}}-d_{\mathrm{nom}}\right)/2$$

The material PMMA has been chosen to establish a setup for EMT that resembles the measurements of breast patients with implanted catheters. Based on Biot‐Savart's law^25^ concerning the magnetic flux, for which the used sensor type is sensible, the expected position shift Δ**p** in the distance r_0_ from the magnet source follows:

(2) $$\frac{\Delta p}{r_{0}}=\left|1-\frac{1}{{}^{3}\sqrt{\mu_{r}}}\right|$$

with $\mu_{r}$ being the relative magnetic permeability of the penetrated medium. Considering water ($\mu_{r}^{\mathrm{water}}$ = 0.999 991) as a medium for the human body, the calculated position shift due to a magnetic field interference is $\frac{\Delta p}{r_{0}}=3\cdot 10^{-6}$. The relative magnetic permeability of PMMA ($\mu_{r}^{\mathrm{PMMA}}\;≲\;1$ deviates insignificantly from water. For both media, the expected position shift Δ**p** therefore is well below (≲ 1‰) the accuracy in air of ≤ 2 mm according to the accompanying calibration protocol (Planar 20‐20 Field Generator, 2015‐01‐26, NDI Europe GmbH, Radolfzell, Germany) of the used EMT system. PMMA thus mimics the human body, so for both phantom and patient measurements correction to account for the measuring medium is omitted.

### 2.C Raw data analysis

In analogy to ongoing patient studies the catheters in the phantom were successively measured by manual displacement of the implant sensor at ~ 40 mm/s thus allowing the measurement of a 12 cm catheter in 3 s. For this displacement velocity and the maximum measuring rate of 40 Hz, we obtained data at an ~ 1 mm interval along a catheter trace. This compromise was found with respect to the upcoming patient study in which a complete measurement of a breast implant with 20 catheters was considered to be tolerated by the patient and staff if completed within 10 min. Each measurement started with the implant sensor fully inserted to the tip‐end of the catheter. After initiating the EM data acquisition, the sensor is held at this position for ~ 2 s until an acoustic signal indicates to the operator that retracting may start. This procedure assures a reliable determination of the tip‐end (see also below).

**Figure 2:**
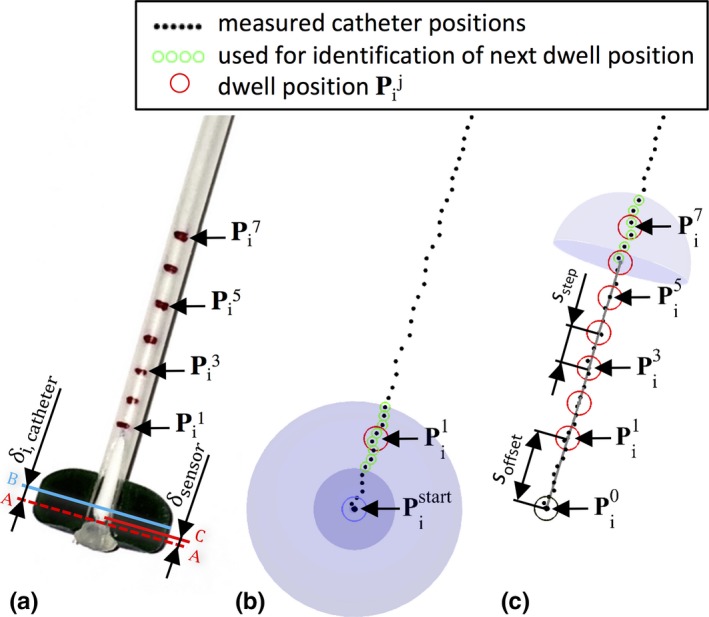
Determination of the dwell position utilizing the measured track. (a) Photo of a catheter‐button configuration. For visualization, the tip‐end area has been cut in half deep and the implant sensor (IS) is inserted up to the catheter stopper position *A*. The marked position *C* at the IS is here the individual measuring location with the distance $\delta_{\mathrm{sensor}}$ to the physical sensor cable tip. The distance from the tip‐end (A) of each catheter *i* to the geometrical button center *B* is referred by *δ*_*i*,catheter_. (b) Beginning on the start position from the measured positions at the catheter's tip‐end, $P_{i}^{\mathrm{start}}$, the first dwell positions $P_{i}^{1}.5\left(j=1\right)$ is identified by the measured positions in a spherical shell. The start position corresponds with the catheter's tip‐end and the button center [see Eqs. (3)–(5)]. (c) According to our treatment planning of breast implants, we set a distance (*s*_offset_) between buttons center $P_{i}^{0}$ and first dwell position $P_{i}^{1}$ of each catheter (*i*). In piecewise linear sections (*s*_step_), the next dwell position is sequentially determined.

The measured EMT‐raw data (see Fig. 2) need to be processed to yield dwell positions. Due to the manual displacement of the implant sensor, the catheter's track is sampled irregularly (i.e., the sensor motion is not constant). In addition, the measured raw position values are not always strictly monotonic with regard to the sensor's motion direction, that is, subsequent values may be in a direction opposite to the movement direction or an accumulation of values can be observed.

For the clinical application, we are interested in EMT reconstructed dwell positions that need to be comparable to the dwell positions defined in treatment planning that is currently based on implant geometry reconstruction on CT images. In addition, an approach to compensate relative movements of the complete implant is required. In clinical practice, such relative movements can be due to an unintended motion of the FG on its position arm or respiratory motion in case of measurements in breast implants. The compensation is achieved by the use of fiducial sensors. In this study, three 6DoF fiducial sensors were fixed on the surface of the quality cross‐check phantom. This mimics the positioning of the fiducial sensors on patients on whom they are taped to the chest. Data from the FS and the IS are acquired simultaneously. For each catheter *i* = {1, …, 8} at each measuring reply *j *= {1, …, 200}, the mean position $p_{i,\mathrm{fiducials}}^{j}$ was calculated from three fiducial sensors.

A position $p_{i,\mathrm{implant}}^{j}$ the implant sensor is corrected using $p_{i,\mathrm{fiducials}}^{j}$ according to:

(3) $$p_{i}^{j}=p_{i,\mathrm{implant}}^{j}-p_{i,\mathrm{fiducials}}^{j}$$

yielding the measured catheter positions $p_{i}^{j}$, compensated for outer movements, and which are further processed to reconstruct the equivalent of dwell positions in analogy to the clinical catheter reconstruction based on CT data.

From the 2 s interval with the implant sensor positioned at the tip‐end of the catheter, labeled $P_{i}^{\mathrm{start}}$ in $R^{3}$) the button center was determined [following Eqs. (3)–(5)]. To be independent of the variable dwell time at the tip‐end, only those positions were taken into account for the mean position, constituting the $P_{i}^{\mathrm{start}}$, which are in a spherical volume with a radius of 0.6 mm. This proved to be a good criterion for separating the amount of measured positions for starting and tracking (see Fig. 2(a) and 2(b)).

Following our clinical routine for breast treatment planning, an offset $s_{\mathrm{offset}}$ of 5 mm is introduced from the button center to the first dwell position $P_{i}^{k}$ (*k *= 1) in $R^{3}$. The next dwell positions $P_{i}^{k}.5\left(k=\left\{2,\ldots ,\;48\right\}\right)$ are determined by the step size of $s_{\mathrm{step}}$ = 2.5 mm. Considering the distance $\delta_{\mathrm{sensor}}$ from the physical sensor cable tip to its individual measuring location (determined by QA routines using the introduced phantom, see Section 2.B) and the distance $\delta_{i,\mathrm{catheter}}$ from the tip‐end of each catheter *i* to its button center (determined by measuring with a precision caliper), the distance *δ*_*i*_ from $P_{i}^{\mathrm{start}}$ to the first dwell position $P_{i}^{1}$ is determined according to:

(4) $$\delta_{i}=s_{\mathrm{offset}}+\delta_{\mathrm{sensor}}+\delta_{i,\mathrm{catheter}}$$

Within the distance range of *δ*_*i*_ ± *s*_step_ the mean position of the located $p_{i}^{j}\left(t_{i}\right)$ was taken to identify the direction vector $u_{i}^{1}$ for the linear equation determining the first dwell position $P_{i}^{1}$ (see Fig. 2(a)):

(5) $$P_{i}^{1}=P_{i}^{\mathrm{start}}+\delta_{i}\cdot u_{i}^{2}$$

The button center $P_{i}^{0}$ was determined into the opposite direction with our clinically usual offset to $P_{i}^{1}$:

(6) $$P_{i}^{0}=P_{i}^{1}-s_{\mathrm{offset}}\cdot u_{i}^{1}$$

The next dwell positions $P_{i}^{k}$ were determined accordingly:

(7) $$P_{i}^{k}=P_{i}^{k-1}+s_{\mathrm{step}}\cdot u_{i}^{k}\;\;\;k=\left\{2,\ldots ,\;48\right\}$$

whereby $P_{i}^{k-1}$ is the current position and $u_{i}^{k}$ is the direction vector, determined by the mean position over the located $P_{i}^{j}$ in the distance of 2 · *s*_*step*_.

### 2.D Registration of dwell positions

In the previous section, the identification of the determined dwell positions $P_{i}^{k}.5\left(k=1,\ldots ,\;48\right)$ of each catheter *i* = {1, …, 8} is described. Based on the known catheter geometry of the used phantom (Section 2.B) the corresponding reference dwell positions $R_{i}^{k}$ are given.

Various automatic registration methods are conceivable 26, 27, 28 and should be compared particularly in terms of their applicability to patient data. In this phantom study, we have selected the rigid coherent point drift registration (CPD),^29^ which only translates and rotates the dataset of the determined dwell positions by an iterative registration routine minimizing the 3D distance of the dwell position pairs. At equal weight for all dwell positions (n = 8 catheters/phantom. 48 dwell positions/catheters = 384 dwell positions/phantom) up to 50 iterations were passed to reach a 12‐digit tolerance criterion (calculation time ~ 0.1 s). The remaining distances $d_{i}^{k}=d\left(P_{i}^{k},R_{i}^{k}\right)$ between the corresponding dwell positions entered as the criterion for the accuracy (Section 2.E.3).

### 2.E Assessment protocols

Based on the reconstructed dwell positions different measurement protocols designed for regular clinical checks were developed using the in‐house developed phantom (Section 2.B). In this context, we report on the precision (Section 2.E.1), on the precision in the presence of motion (Section 2.E.2) and on the accuracy of the EMT‐based dwell position definition (Section 2.E.3). To assess the clinical applicability of EMT during breast brachytherapy, the measurements were conducted on the CT scanner table in the CT room, on the treatment table in the HDR treatment room and on the patient bed in one of our PDR treatment rooms.

For each measurement used in the study, the setup is displayed in Fig. 1(c). The setup allows varying the distance *d*_FG_ from the FG to the catheter center located in the phantom. In addition, by lifting the phantom, the distance *d*_table_ to the table (or bed) top can be varied, for example, to investigate the influence of ferromagnetic materials in the table (or the patient bed). FG distances of *d*_FG_ ≅ 100, 200 and 300 mm were studied by adjusting the positioning arm holding the FG. On purpose, these were not adjusted precisely but rather used as a clinical reference point and variations are expected since also in clinical routine an exact positioning of the FG at these reference distances is not feasible. The distance *d*_table_ was varied to 0, 30, 60, 80, 120 mm by placing Styrodur^®^ pads ($\mu_{r}^{\mathrm{Polystyrol}}\approx 1-8\cdot 10^{-6}$,^30^ according to Eq. (1), $\frac{\Delta p}{r_{0}}<2.7\cdot 10^{-6}\;\text{mm}$) under the phantom. The setup was randomly positioned on the table at locations corresponding to the clinical set‐up for EMT of an implant in the left or right breast of a patient, thus including potential influence of ferromagnetic table components such as metal struts, screws or bolts.

#### 2.E.1 Precision

The term precision is used (in accordance with ISO/IEC Guide 99:2007)^31^ as “closeness of agreement between measured positions obtained by replicate measurements under specified conditions”. The precision *f*_*p*_ = *f*_*p*_(*d*_FG_, *d*_table_) was determined by measuring the variation in the stable position with the implant sensor fully inserted into a catheter (here, *i* = 7) with position correction based on the fiducial sensors as described previously (Section 2.C). The combinations of the above‐specified five distances *d*_FG_ and three distances *d*_table_ were investigated on all of the three different clinical tables. For each combination, six phantom measurements of 400 measured positions per catheter were recorded.

According to the Shapiro‐Wilk test ~ 10 % of the measurements were not normally distributed (H = 0, *P* = 0.05, n ~ 1/10). The measured data were thus mainly analyzed based on the 50th (median), 95th and 99th percentile. As suggested by Nixon et al.,^22^ the median and the 95th percentile were fitted by adjusting *k* to $f_{p}=k\;d_{\mathrm{FG}}^{4}{\left(d_{\mathrm{metal}}+d_{\mathrm{table}}\right)}^{-3}\;d_{\mathrm{table}}^{-3}$, whereby *d*_metal_ = 60 mm over all settings, used for estimated distance *d*_metal_ +* d*_table_ from the sensor to metal objects in the tables/bed considering the half phantom height (Section 2.B) and a supporting cushion or mattress.

In addition to these short‐term measurements, multiple long‐term measurements (30 min) have been recorded. Those measurements were performed under equal conditions on the HDR treatment table at *d*_FG_ ≅ 200 mm and *d*_table_ ≅ 120 mm. The initial condition was that the EMT system was six hours unplugged in the air‐conditioned treatment room at 22 °C. The first 5 minutes should quantify the recommended warm‐up time from the manufacturer. Until the 20th minute, the long‐term stability was observed corresponding to the double of the typical time duration of patient measurements. Afterwards, potential sources of influence on the precision of EM tracking were initiated (details in Table 1).

**Table 1:** Potential sources of influence examined in long‐term (30 min) measurements

Label	Time period (min)	Potential sources of influence in the HDR treatment room
C	20th–21st	Switch on/off of different light switches, plug in/out the power supply of the laptop
D	21st–22nd	Operate different servomotors of the treatment table
E	22nd–23rd	Move the afterloader close to the treatment table and transfer cable in the tracking field
F	23rd–24th	Move the metallic side table close to the treatment table
G	24th–25th	Move the on the ceiling fixed junction box close to the FG
H	25th–26th	Move through the tracking field different ballpoints, metal pins and metallic catheters
I	26th–27th	Bring an active mobile phone close to the tracking field and call it
J	27th–28th	Bring an active clinical DECT phone close to the tracking field and call it
K	28th–29th	Switch on/off the Wi‐Fi of the measurement laptop
L	29th–30th	Sudden shaking of the FG in different directions by pushing on the locked position arm

#### 2.E.2 Dynamic precision

In clinical use, the fiducial and implant sensors will be influenced by respiratory motion. Precision measurements were also conducted under these circumstances by sliding the phantom setup on a 1D motion drive 32 (QRM GmbH, Möhrendorf, Germany) perpendicular, parallel, and toward the FG, that is, 1D movements in all three spatial directions. Relative movement velocities *v*_phantom_ = (10, 20, 30, 40, 50, and 60 mm/s) were studied by moving the table back‐and‐forth during the 1 min measurement interval. The measurements were performed only on the HDR treatment table at 200 mm ≤ *d*_table_ ≤ 300 mm and 150 mm ≤ *d*_FG_ ≤ 250. For each setup combination, three measurements were recorded. To exclude accelerations and reversals of motion from raw data, the *v*_phantom_ ± 2 mm/s were used. Analysis of the remaining data under the influence of motion was performed as described in the previous section.

#### 2.E.3 Accuracy

The term accuracy was used (in accordance with ISO/IEC Guide 99:2007) 31 as “the closeness of agreement between a measured quantity and a true quantity value of a measurement”. The determined dwell positions were used as “measured quantity”, the reference dwell positions based on the fixed catheters guided in the phantom with known geometry were interpreted as “true quantity value”. This definition of the accuracy encompasses the interpretation of the measured positions including the registration as described in Section 2.C.

### 2.F Error detection

One of the goals for the introduction of EMT in brachytherapy is reliable error detection. To show the feasibility of error detection based on registering the nominal (planned) and daily (measured) implant geometry as described in Section 2.D we selected and simulated two potential error causes: (a) the swapping of two catheters (*i* = 6 and 7) and (b) the displacement ∆*l* = 0, 1, 2, 3, 4, 5, and 6 mm of a single catheter (*i *= 6) relative to the remaining catheters.

According to the assessment protocol of the measurement accuracy (Section 2.E.3), the measurements were performed three times for the both error simulation cases on the HDR treatment table at *d*_FG_ ≅ 200 mm and *d*_table_ ≅ 120 mm. The distances $d_{i}^{k}$ between the reference and the determined positions of each catheter (*i* = {1, …, 8}) were considered with respect to the first dwell positions (*k* = 1).

## 3 Results

### 3.1 Precision

To assess the precision of the used EMT at the different settings 50th (median), 95th and 99th percentile were analyzed on the three different clinical tables. Based on grouping the following results were found: On the CT table, the 95th percentile reached up to 0.12 mm (0.34 mm for 99th percentile; 0.03 mm for median) over all distances FG to catheter (*d*_FG_) and heights of Styrodur^®^ pads (*d*_table_). For measurements at *d*_FG_ ≅ 100 mm, the EMT system failed due to a high tracking error signaled by the EMT system most likely due to interferences with the metallic components of the table. The results for the HDR treatment table yielded the 95th percentile at 0.13 mm (0.20 mm for 99th percentile; 0.04 mm for median), and for the patient bed in the PDR treatment room (“PDR patient bed”) a maximal precision value of 0.17 mm (95th percentile) was found (0.30 mm for 99th percentile; 0.04 mm for median).

**Figure 3:**
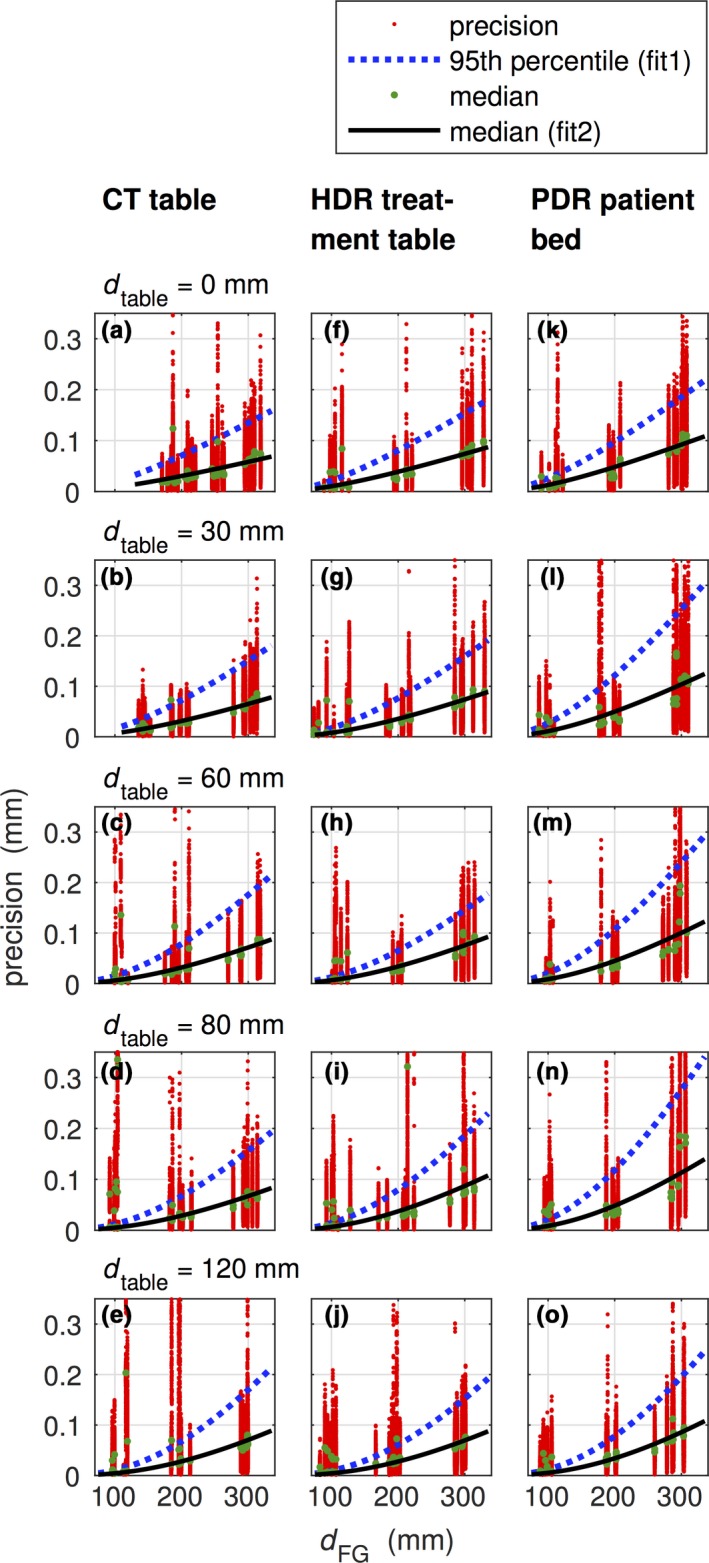
Precision data determined for the CT table (a–e), the HDR treatment table (f–j) and the PDR patient bed (k–o) for different heights of Styrodur^®^ pads (0 mm ≤ *d*_table_ ≤ 120 mm). The distances between the FG and the phantom were approximately around *d*_FG_ = 100 mm, 200 mm, and 300 mm. 95th percentile (fit1) and median (fit2) were fitted according to $f_{p}=k\;d_{\mathrm{FG}}^{4}{\left(d_{\mathrm{metal}}+d_{\mathrm{table}}\right)}^{-3}\;d_{\mathrm{table}}^{-3}$ following the approach of Nixon et al.^22^

Figure 3 shows the precision values for the different settings. The data for median and 95th percentile are fitted following the approach of Nixon et al.^22^ Details for the different settings can be found in Table S1 (available in the Supplementary Materials file available on the JACMP website at www.jacmp.org).

**Figure 4:**
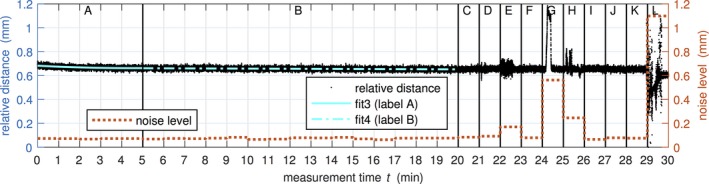
Example of the long‐term measurements. The various actions in the highlighted time intervals (label A–L) are described in Section 2.E.1 and Table 1. The measured distance to the coordinate origin defined by the fiducials is plotted on the left axis, whereby the minimum is shifted to zero millimeter. The maximum fluctuation per minute section is plotted as noise level on the right axis.

Figure 4 shows a typical result of a long‐term measurement to examine potential sources of influence. Within the recommended warm‐up time of 5 min (label A) a drift of the relative distance of 0.02 mm was observed. The linear fit from 5th to 20th minute (label B) showed a drift of 0.01 mm. The noise level was stable at 0.08 ± 0.01 mm for the first 20 minutes in which the measurement was not disturbed. From the sources of interference introduced beyond 20 min (see Table 1) only the following ones showed an effect: Metallic components close to the tracking field (label E and G, noise level of 0.17 mm and 0.57 mm, respectively) and shaking of the FG (label L, noise level of 1.1 mm).

### 3.2 Dynamic precision

**Figure 5:**
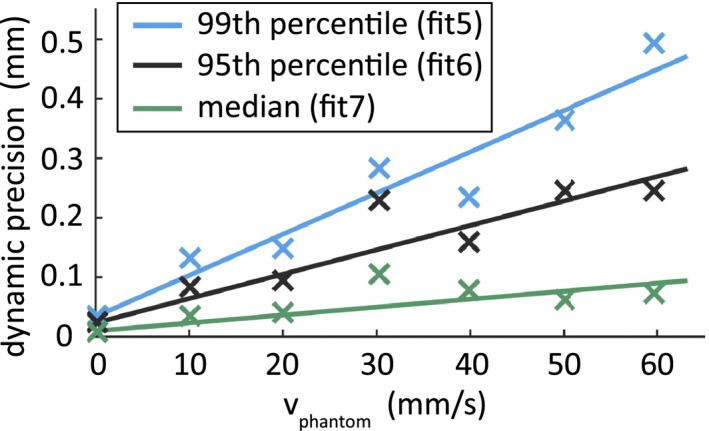
Dynamic precision. Linear fit functions over the median, 95th and 99th percentile of the movement velocities from 0 mm/s to 60 mm/s. The constant coefficient was fixed to the determined value at rest (0 mm/s).

Further analysis of the precision under the condition of a relative movement of the FG to the phantom showed that the used EMT system can follow our sensors within a precision of 0.27 mm (95th percentile) at the maximum investigated movement velocity of 60 mm/s. The dependency of median, 95th and 99th percentile on the movement velocity is depicted in Fig. 5. Since the different spatial directions did not show an influence on the precision, the data were gathered for each setting of the movement velocity.

### 3.3 Accuracy

Accuracy was studied in the same settings as precision. The median over each catheter measurement at the CT table at *d*_table_ = 120 mm was 0.49 mm, 0.58 mm, and 0.66 mm for *d*_FG_ = 100 mm, 200 mm, and 300 mm, respectively. Under the same conditions, at the HDR treatment table the median was 0.42 mm, 0.52 mm, and 0.63 mm and at the PDR patient bed the median was 0.42 mm, 0.59 mm, and 0.75 mm, respectively. With decreasing distance *d*_table_ the median increased approximately linearly. In the CT room, the small table distances resulted in a reduced accuracy [4.04 mm (95th percentile) for *d*_table_ ≤ 60 mm].

At the maximum investigated *d*_table_ of 120 mm the maximum 95th and 99th percentile were at the CT table 1.07 mm and 1.49 mm, at the HDR treatment table 0.98 mm and 1.25 mm and at the PDR patient bed 1.15 mm and 1.49 mm, respectively.

**Figure 6:**
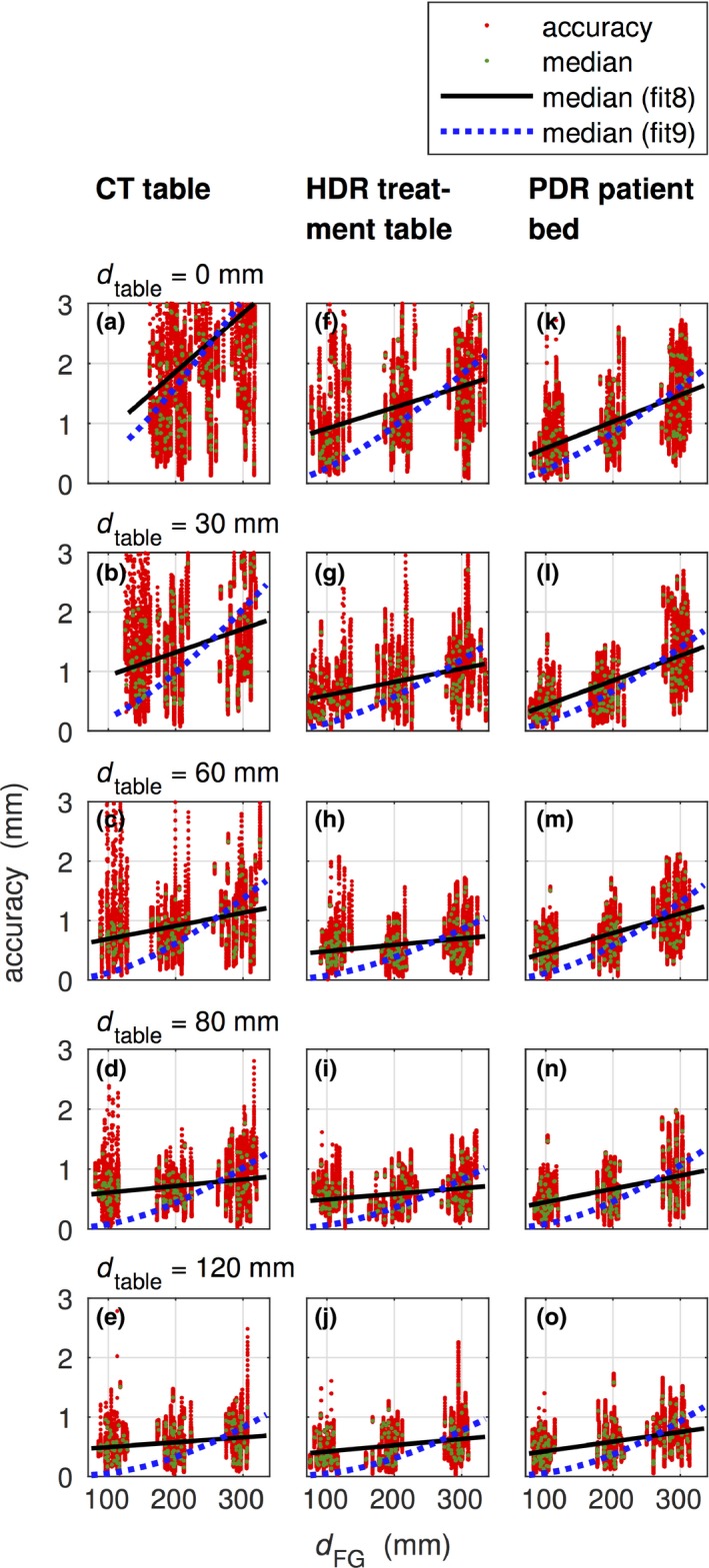
Accuracy to the CT table (a–e), HDR treatment table (f–j) and PDR patient bed (k–o) for different heights of Styrodur^®^ pads (0 mm ≤ *d*_table_ ≤ 120 mm) and the FG distances *d*_FG_ around the 100 mm, 200 mm, and 300 mm. The fit function fit8 followed a linear polynomial and fit9 the approach of Nixon et al.^22^ on the median values per catheter measurement. For visualization purpose, we displayed only accuracy values up to 3 mm.

The linear polynomial (fit8) and the approach of Nixon et al.^22^ (fit9) are fitted to the median data (see Fig. 6). Based on these fits accuracy data for different settings are provided in Table S3 (in the supplementary materials).

### 3.4 Error detection

Swapping of catheters was detected (case a). The deviation at the 1st dwell positions are close to the measured geometrical distance (= 20.0 mm), see Table 2. The gradual displacement of a single catheter (case b) showed that the geometrical shifts ∆*l* are close to the determined distances, see Table 3. Over all, both examples for error detection were below the determined accuracy (95th percentile of 0.83 mm).

**Table 2:** Example for error detection by analyzing the distances $d_{i}^{k}$ between the reference and the determined positions of each catheter *i* (*i* = 1, 2, …, 8). In the example, the first dwell position (*k *= 1) is analyzed and catheters *i *= 6 and 7 were swapped. The measured geometrical distance between those catheters is 20.0 mm

i	1	2	3	4	5	6	7	8
$d_{i}^{k}$ (mm)	0.40	0.59	0.68	0.65	0.31	20.07	20.06	0.20

**Table 3:** Example for error detection by displacement (∆*l* = 0, 1, 2, 3, 4, 5, and 6 mm) of catheter *i* = 6. The matrix lists the distance $d_{i}^{k=1}$ between the reference and the determined positions of each catheter for the first dwell position in dependence of ∆*l* in millimeter

∆*l* (mm)	Catheter *i*							
	1	2	3	4	5	6	7	8
0	0.47	0.63	0.77	0.71	0.28	0.17	0.32	0.20
1	0.49	0.67	0.71	0.73	0.23	1.09	0.37	0.12
2	0.31	0.52	0.76	0.75	0.36	2.16	0.48	0.19
3	0.40	0.63	0.75	0.78	0.25	3.14	0.39	0.12
4	0.33	0.52	0.68	073	0.37	4.34	0.51	0.22
5	0.37	0.57	0.70	0.74	0.27	5.27	0.37	0.12
6	0.46	0.71	0.72	0.34	0.34	6.09	0.37	0.08

## 4 Discussion

A number of studies showed that EMT is a viable approach for quality assurance and implant tracking in brachytherapy.^19^ This study is the technical foundation of a recently initiated clinical investigation to study potential changes of the implant geometry in interstitial HDR brachytherapy of breast cancer by EMT. To allow drawing conclusions from this clinical investigation, a sound data acquisition and reconstruction workflow had to be established aiming at a precision and accuracy level sufficient for that purpose.

The chosen approach for this investigation is thus fully oriented toward the clinical setting. EMT acquisition workflows were designed for the clinical use. This requirement includes mobile equipment that can easily be adapted for an individual patient on the different clinical tables (in the CT, HDR, and PDR room). But also quick acquisition times for each catheter to allow the measurement of 20 catheters within 10 min. This lead us to continuous EMT acquisitions at ~ 40 mm/s and 5 s per catheter (= 100 s) such that 10 s for each catheter change in the implant sensor (= 200 s) and 5 min for setup of the system and positioning of the fiducial sensors still allows to meet the 10 min constraint. In the reported measurements, the phantom was handled like a patient, that is, deliberate but not fully controlled positioning on the tables and in the field‐of‐view of the field generator. This leads to varying vicinity, for example, to metal bolds in the HDR table, and slight deviation to target positions *d*_FG_. Depending on the experience in the upcoming months an EMT‐designed environment might be established including, for example, dedicated treatment tables and permanent installation of the field generator. Since such installations are not present in the current clinical investigation they were also not introduced for this study.

EMT measurements do not provide direct determination of the dwell positions. In addition, the high frame rate allows to average raw data to reduce the sensitivity of determined dwell positions on the precision of the individual measurement points. Each group chooses different approaches for data acquisition and processing. Damato et al. measure step‐by‐step in 1 cm intervals with mean acquisition times of 4.7 s per step.^23^ For their phantom with 15 catheters and on average 14.3 steps per catheter this leads to measurement times of 1008 s plus overhead. Presumably, the investigation did not focus on optimization of measurement speed but at least in our clinic such measurement times would not be tolerated on a fraction‐by‐fraction level. Raw data for each step are averaged and dwell positions interpolated at the needed distance. Bharat et al. chose an approach similar to ours.^10^ The sensor is retracted starting from the catheter tip with acquisition times of 5 – 10 s per catheter. Data are registered with the coordinate system of trans‐rectal ultrasound and CT by reference sensors similar to the fiducial sensors used in this study. Coordinate systems are then matched by a rigid registration. Also, in the methodology of Poulin et al., in which the noisy raw data is smoothed aside from the tip‐position of the catheter and reconstruct trajectory points each 5 mm, catheter acquisition times of 10 s are feasible.^21^

Matching EMT data with other imaging data is one of the key challenges that need to be addressed. A comprehensive overview of the currently used techniques is provided by Zhou et al.^19^ For rigid objects such as the phantoms used in accuracy studies registration between EMT and reference data are a feasible alternative even though potentially introduced systematic shifts could be canceled out by the registration. Deliberately placed fiducial sensors, for example, on the three orthogonal faces of a phantom, can overcome this limitation. In patients, matching of coordinate systems depends on the application. Many potential errors, such as the swap of catheters, a catheter displacement, or wrong catheter reconstruction by intersecting between catheters can be detected via intra‐implant distances. These distances are independent of the coordinate system. Alternatively, a registration of the coordinate system of treatment planning and the one of EMT can be performed without using fiducial sensors. The error detection sensitivity and specificity then mainly depends on the EMT accuracy as reported by Damato et al.^23^ In section 3.D, we showed an example of registration based error detection that could detect catheter swaps and shifts down to Δl=1mm. In case, the implant is compared to anatomic structures of the patient, for example, to perform dose calculation, or if inter‐fractional changes of the patient's anatomy with respect to CT scan are of interest, registration needs landmarks visible in both techniques. As recently reported by Zhou et al., three fiducial sensors are a starting point for rigid registration of the coordinate systems with uncertainties related to reproducibly placing the fiducials.^19^ A combination of EMT with surface imaging could improve the registration and also be sensitive to potential non‐rigid inter‐fractional changes of the patient's anatomy, for example, changes in breast posture. Various surface imaging systems are available mainly for patient positioning in external beam radiation therapy.^33^, ^34^, ^35^ CT‐extracted surfaces could be used as a reference such that daily measured patient surfaces in combination with, for example, IR‐reflective fiducials on the EMT‐fiducial sensors would allow a registration of EMT and CT‐surface on a daily level.

A necessity for all applications of EMT is a precisely and accurately working system. Sections 3.A–C focused on this issue as did several other initial reports on EMT in brachytherapy. NDI specifies for the used 5DoF sensor a precision of 0.48 mm RMS (1.00 mm at 95 % confidence interval (CI)) and an accuracy of 0.70 mm (at 1.40 mm 95 % CI). For the used 6DoF sensors, a precision of 0.30 mm RMS (0.70 mm at 95 % CI) and an accuracy of 0.48 mm (at 0.88 mm 95 % CI) can be taken from the technical specification for the employed EMT system. As shown in section 3.A, we determined 95th percentiles of 0.12 mm, 0.13 mm and 0.17 mm (mean ± SD of 0.05 ± 0.07, 0.05 ± 0.05, and 0.06 ± 0.06 mm) for CT, HDR, and PDR room, respectively, for fiducial sensor corrected reading of the steady implant sensor. This quantity is comparable to the relative accuracy of the study of Bharat et al. who also used the Aurora system and reported a mean translation relative accuracy of 0.19 mm (maximum 1.45 mm). We could observe precision values increase proportionally to $d_{\mathrm{FG}}^{4}\cdot d_{\mathrm{table}}^{-3}$ as derived by Nixon et al.^22^ with larger deviations from the fitted curve at small field generator distances ($d_{\mathrm{FG}}\;<\;120\;\text{mm}$). The precision values of all studied *d*_FG_ to *d*_table_ combinations on all three clinical tables are < 0.34 mm for the 99th percentile, that is, lower than the source precision of ± 1 mm specified by the manufacturers.^9^ Only metallic objects such as a junction box with metallic components lowered from the ceiling or sudden shaking of the FG influenced the precision.

Since EMT will be used for breast implant measurements, the reading of the implant sensor needs to be corrected with respect to overlaid breathing motion. Currently, the mean of the three fiducial sensor's position is used for that purpose, but more sophisticated correction protocols are likely needed since the mean of the fiducial sensors will most likely not precisely reflect the motion influence at the position of the implant sensor. In section 3.B, the precision under the influence of motion was determined. We observed a linear dependency on the motion velocity. But even at 60 mm/s, which is more than the expected velocities due to breathing motion,^36^ the 95th percentile reaches only 0.19 mm which is tolerable for the clinical goals. In previous studies, for example, of Nafis et al.^37^ higher values were reported (0.54 mm at 50 mm/s) potentially due to more irregular motion patterns.

With respect to accuracy, we chose a precision built phantom as ground truth to be independent of the limited resolution of imaging data and/or the limited calibration of the imaging device. The effect of such influence was reported by Poulin et al.^21^ They determined that the identification error of the sensor tip changed from 0.69 mm to 1.08 mm when the ground truth changed from μCT to CT, respectively. The accuracy was more influenced by the distance from the bed/table (*d*_table_) than the precision (see Fig. 6). Especially on the CT table, measurements in a range of 0 ≤ *d*_table_ ≤ 60 mm are not recommended since the accuracy deviates heavily (median: 1.30 mm, RMSE: 1.16 mm, 95th and 99th percentile: 4.0 mm and 5.64 mm) over all ranges of studied field generator distance *d*_FG_. At ranges *d*_table_ ≥ 80 mm are distributions are narrower with an accuracy of median: 0.62 mm, RMSE: 0.33 mm, 95th and 99th percentile: 1.28 mm and 1.61 mm that is compatible with the expected level. The table distance also influences the accuracy on HDR treatment table and PDR patient bed but there the influence is lower: For *d*_table_ ≥ 80 mm, we determined accuracies of median: 0.52 mm, RMSE: 0.29 mm, 95th and 99th percentile: 1.07 mm and 1.37 mm on the HDR treatment table, and of median: 0.56 mm, RMSE: 0.35 mm, 95th and 99th percentile: 1.30 mm and 1.72 mm on the PDR patient bed, respectively. The NDI technical specifications report an approximating constant accuracy in the studied field generator distances in an undisturbed environment. Nixon et al.^22^ show that the positioning error is proportional to $d_{\mathrm{FG}}^{4}$ and Zhou et al. show that the combination of large field generator distances and interferences of a typical brachytherapy environment yields the largest error.^19^, ^20^ The data at $d_{\mathrm{table}}=120\;\text{mm}$ and over all *d*_FG_ reported in Fig. 6 neither show a constant behavior of the accuracy dependence on *d*_FG_ (RMSE: 0.30 mm (CT), 0.36 mm (HDR), and 0.30 mm (PDR)) nor a proportionality to $d_{\mathrm{FG}}^{4}\cdot d_{\mathrm{table}}^{-3}$ [RMSE: 0.40 mm (CT), 0.36 mm (HDR) and 0.37 mm (PDR)]. They are best described by a linear dependency [RMSE: 0.26 mm (CT), 0.25 mm (HDR) and 0.26 mm (PDR)]. All clinical tables yielded sufficiently low accuracy values for application in breast cancer patients treated in the supine position. Then the implant is positioned at *d*_table_ > 120 mm and the field generator can easily be placed close to the breast ($d_{\mathrm{FG}}\;≲\;200\;\text{mm}$. According to Kolkman‐Deurloo et al.^38^ the 95th percentile of the reconstruction error should be ≲2mm which is in line with our clinical tolerances. In case other patient cohorts with positioning protocols at small distances to bed/table are measured by EMT environment specific system calibration seems to be an option.^39^ A potentially easier alternative is the artificial enlargement of *d*_table_, for example, by foam‐pads beneath the patient, if EMT‐compatible tables are not available.

Aside from an application in error detection as initially shown in section 3.D EMT is also a promising tool for implant reconstruction or adaptive treatment schemes. The level of accuracy corresponds to currently accepted catheter reconstruction accuracies in the community 9, 40 and thus clinical tests are the next step to evaluate workflow feasibility and clinical benefit.

**Figure 7:**
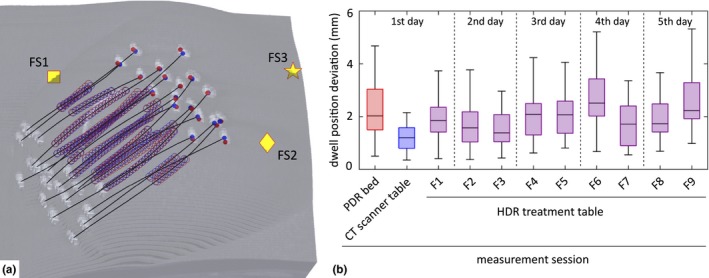
(a) Superposition of the clinically used treatment planning of the source dwell positions (blue circles) with the corresponding dwell positions (red circles) determined from the EMT measurement in a patient's breast implant. The CT‐based representation shows reconstructed catheters (black lines) between the catheter's button centers. The symbols labeled FS1, FS2 and FS3 show the position of the three fiducial sensors placed on the patient's skin. (b) Boxplots show the dwell position deviation from measurement sessions of the HDR‐iBT breast treatment during 5 days.

Figure 7 gives an insight into the clinical application of the above‐investigated concept. The results in different clinical environments were in median in the range of 1–2 mm (see boxplot). The lower deviation between the CT‐based treatment planning (used as reference data) and the corresponding dwell positions determined from the EMT measurements on the CT scanner table are expected, because they were measured back‐to‐back without moving the patient. The results to date indicate that the deployed motion compensation is useful when applied to patient data. Clinical implementation on a daily level needs further investigations with respect to reference data in case error or uncertainty detection is the aim of the implementation. A smooth implementation of such a system into the clinical workflow, that is, proper placement of the field generator, automatic recording of EMT data, for example, by incorporating the sensor into an afterloader are desired. Furthermore, appropriate quality assurance methods of the EMT system including site‐checks to spot metallic components potentially influencing the EMT acquisition need to be developed.

## 5 Conclusions

EMT is a precise and accurate solution to determine dwell positions of the measured implant in the typical clinical setting of HDR and PDR brachytherapy environments for iBT of the breast. Precision values (95th percentile) below 0.12 mm (CT table), 0.13 mm (HDR table) and 0.17 mm (PDR bed) were determined for all field generator distances and table/bed distances in all three clinical tables. The accuracy showed a dependence on the table distance especially on the CT table, where the accuracy was 4.04 mm (95th percentile) for *d*_table_ ≤ 60mm. For larger table distances (*d*_table_ = 120 mm) on the CT and HDR table as well as on PDR bed the accuracy was 1.07 mm, 0.98 mm, and 1.15 mm (95th percentile), respectively, and thus comparable to the accuracies achieved with alternative EMT solutions. Measurement times of typical implant geometries can be performed in 5–10 min depending on the technical implementation in the brachytherapy environment. Studies evaluating the potential of EMT in clinical practice are indispensable. Therefore, measurements of the in‐situ implant geometry in HDR breast patients have been initiated at the university clinic in Erlangen.

## Supporting information


**Table S1.** Precision according to the fit functions fit1 and fit2, which are based on Nixon et al.^22^ (see Fig. 3 over median (fit2) and 95th percentile (fit1): $f_{p}=k\;d_{\mathrm{FG}}^{4}{\left(d_{\mathrm{metal}}+d_{\mathrm{table}}\right)}^{-3}\;d_{\mathrm{table}}^{-3}$, using *d*_metal_ = 60 mm
**Table S2.** Dynamic precision at different movement velocities. Values based on linear fit functions (fit5, fit6 and fit7) over the 99th, 95th, and 50th percentile (see also Fig. 5)
**Table S3.** Accuracy on median data according to the linear polynomial fit8: $f_{a}=p_{1}\;d_{\mathrm{FG}}+p_{2}$ and to the approach of Nixon et al.^22^ fit9 (see also Fig. 6): $f_{a}=k\;d_{\mathrm{FG}}^{4}{\left(d_{\mathrm{metal}}+d_{\mathrm{table}}\right)}^{-3}\;d_{\mathrm{table}}^{-3}$, using *d*_metal_ = 60 mm.
