# Supplementary material for: Electromagnetic tracking (EMT) technology for improved treatment quality assurance in interstitial brachytherapy
Source: J Appl Clin Med Phys. 2017 Jan 19;18(1):211–22. doi: 10.1002/acm2.12021 (PMC5689872; doi:10.1002/acm2.12021)
Supplement: Supplementary file 1 — Table S1. Precision according to the fit functions fit1 and fit2, which are based on Nixon et al.22 (see Fig. 3 over median (fit2) and 95th percentile (fit1): fp=kdFG4(dmetal+dtable)−3dtable−3, using d metal = 60 mm Table S2. Dynamic precision at different movement velocities. Values based on linear fit functions (fit5, fit6 and fit7) over the 99th, 95th, and 50th percentile (see also Fig. 5) Table S3. Accuracy on median data according to the linear polynomial fit8: fa=p1dFG+p2 and to the approach of Nixon et al.22 fit9 (see also Fig. 6): fa=kdFG4(dmetal+dtable)−3dtable−3, using d metal = 60 mm. [file ACM2-18-211-s001.docx]

# **Supplementary Material**

**Table S1.** Precision according to the fit functions fit1 and fit2, which are based on Nixon et al. ^(22)^ (see Fig. 3 over median (fit2) and 95^th^ percentile (fit1): $f_{p}=k d_{\mathrm{FG}}^{4} {(d_{\mathrm{metal}}+d_{\mathrm{table}})}^{-3} d_{\mathrm{table}}^{-3}$, using *d*_metal_ = 60 mm

|  |  | CT table | | HDR treatment table | | PDR bed | |
| --- | --- | --- | --- | --- | --- | --- | --- |
| *d*_table_ (mm) | *d*_FG_ (mm) | fit2: median (mm) | fit1: 95^th^ perc. (mm) | fit2: median (mm) | fit1: 95^th^ perc. (mm) | fit2: median (mm) | fit1: 95^th^ perc. (mm) |
|  | 100 | Failure of EMT | | 0.01 | 0.02 | 0.01 | 0.03 |
| 0 | 200 | 0.03 | 0.07 | 0.04 | 0.08 | 0.05 | 0.10 |
|  | 300 | 0.06 | 0.13 | 0.07 | 0.15 | 0.09 | 0.19 |
|  | 150 | 0.01 | 0.02 | 0.01 | 0.02 | 0.01 | 0.03 |
| 30 | 200 | 0.03 | 0.07 | 0.04 | 0.08 | 0.05 | 0.12 |
|  | 300 | 0.07 | 0.15 | 0.07 | 0.16 | 0.10 | 0.25 |
|  | 100 | 0.01 | 0.02 | 0.01 | 0.01 | 0.01 | 0.02 |
| 60 | 200 | 0.03 | 0.08 | 0.03 | 0.07 | 0.04 | 0.11 |
|  | 300 | 0.07 | 0.18 | 0.08 | 0.15 | 0.10 | 0.24 |
|  | 100 | 0.01 | 0.01 | 0.01 | 0.01 | 0.01 | 0.02 |
| 90 | 200 | 0.03 | 0.07 | 0.04 | 0.08 | 0.05 | 0.12 |
|  | 300 | 0.07 | 0.16 | 0.09 | 0.19 | 0.11 | 0.28 |
|  | 100 | 0.00 | 0.01 | 0.00 | 0.01 | 0.01 | 0.01 |
| 120 | 200 | 0.03 | 0.07 | 0.03 | 0.06 | 0.03 | 0.08 |
|  | 300 | 0.07 | 0.17 | 0.07 | 0.15 | 0.09 | 0.20 |

**Table S2.** Dynamic precision at different movement velocities. Values based on linear fit functions (fit5, fit6 and fit7) over the 99^th^, 95^th^ and 50^th^ percentile (see also Fig. 5)

| Velocity (mm/s) | 0 | 10 | 20 | 30 | 40 | 50 | 60 |
| --- | --- | --- | --- | --- | --- | --- | --- |
| Median (mm) | 0.01 | 0.02 | 0.04 | 0.05 | 0.06 | 0.08 | 0.09 |
| 95^th^ percentile (mm) | 0.02 | 0.06 | 0.11 | 0.15 | 0.19 | 0.23 | 0.27 |
| 99^th^ percentile (mm) | 0.03 | 0.10 | 0.17 | 0.24 | 0.31 | 0.38 | 0.45 |

**Table S3.** Accuracy on median data according to the linear polynomial fit8: $f_{a}=$ *p*_1_ *d*_FG_ + *p*_2_ and to the approach of Nixon et al. ^(22)^ fit9 (see also Fig. 6): $f_{a}=k d_{\mathrm{FG}}^{4} \left( d_{\mathrm{metal}}+d_{\mathrm{table}} \right)^{-3} d_{\mathrm{table}}^{-3}$, using *d*_metal_ = 60 mm.

|  |  | CT table | | HDR treatment table | | PDR bed | |
| --- | --- | --- | --- | --- | --- | --- | --- |
| *d*_table_ (mm) | *d*_FG_ (mm) | fit 8 (mm) | fit 9 (mm) | fit 8 (mm) | fit 9 (mm) | fit 8 (mm) | fit 9 (mm) |
|  | 100 | Failure of EMT | | 0.92 | 0.26 | 0.59 | 0.23 |
| 0 | 200 | 1.86 | 1.61 | 1.27 | 0.95 | 1.03 | 0.85 |
|  | 300 | 2.84 | 3.07 | 1.62 | 1.82 | 1.48 | 1.61 |
|  | 150 | 0.93 | 0.22 | 0.60 | 0.13 | 0.43 | 0.15 |
| 30 | 200 | 1.32 | 0.98 | 0.82 | 0.57 | 0.85 | 0.67 |
|  | 300 | 1.71 | 2.05 | 1.05 | 1.20 | 1.27 | 1.40 |
|  | 100 | 0.69 | 0.12 | 0.48 | 0.07 | 0.46 | 0.11 |
| 60 | 200 | 0.91 | 0.61 | 0.59 | 0.38 | 0.79 | 0.58 |
|  | 300 | 1.13 | 1.37 | 0.70 | 0.85 | 1.12 | 1.31 |
|  | 100 | 0.61 | 0.08 | 0.49 | 0.06 | 0.45 | 0.08 |
| 90 | 200 | 0.72 | 0.44 | 0.58 | 0.35 | 0.67 | 0.46 |
|  | 300 | 0.83 | 1.02 | 0.68 | 0.82 | 0.89 | 1.06 |
|  | 100 | 0.49 | 0.05 | 0.42 | 0.05 | 0.42 | 0.06 |
| 120 | 200 | 0.58 | 0.33 | 0.52 | 0.31 | 0.59 | 0.37 |
|  | 300 | 0.66 | 0.82 | 0.63 | 0.77 | 0.75 | 0.93 |
